# Sex differences in immune responses to SARS-CoV-2 in patients with COVID-19

**DOI:** 10.1042/BSR20202074

**Published:** 2021-01-29

**Authors:** Guolian Zhao, Yazhou Xu, Jing Li, Xiaoli Cui, Xiaowen Tan, Hongyue Zhang, Liyun Dang

**Affiliations:** 1 Department of Laboratory Medicine, Xi’an Chest Hospital, Xi’an, China; 2 Department of Etio-biology, Southern Medical University, Guangzhou, China; 3 Reproductive Medical Center, The Sixth Affiliated Hospital of Sun Yat-Sen University, Guangzhou, China; 4 Key Laboratory of Carcinogenesis and Translational Research (Ministry of Education/Beijing), Laboratory of Molecular Oncology, Peking University Cancer Hospital and Institute, Beijing, China

**Keywords:** biomarkers, COVID-19, estrogen, immune responses, sex-specific

## Abstract

Millions of people infected by severe acute respiratory syndrome coronavirus 2 (SARS-CoV-2) have been diagnosed with coronavirus infectious disease 2019 (COVID-19). The prevalence and severity of COVID-19 differ between sexes. To explain these differences, we analyzed clinical features and laboratory values in male and female COVID-19 patients. The present study included a cohort of 111 people, i.e. 36 COVID-19 patients, 54 sex- and age-matched common viral community-acquired pneumonia (CAP) patients, and 21 healthy controls. Monocyte counts, lymphocyte subset counts, and alanine aminotransferase (ALT), aspartate aminotransferase (AST), and C-reactive protein (CRP) levels in the peripheral blood were analyzed. Higher Acute Physiology and Chronic Health Evaluation II (APACHE II) scores, monocyte counts, and CRP and ALT levels were found in male COVID-19 patients. Decreased lymphocyte subset counts and proportions were observed in COVID-19 patients, except for the CD3^+^ and CD8^+^ T cell proportions. The lower CD4^+^ T cell proportions and higher CD8^+^ T cell proportions were observed in male and severe COVID-19 patients and the differences were independent of estrogen level. The CD4^+^ T cell proportion was negatively associated with the CD8^+^ T cell proportion in male COVID-19 patients; this correlation was non-significant in females. Our work demonstrates differences between sexes in circulating monocyte counts and CD4^+^ T cell and CD8^+^ T cell proportions in COVID-19 patients, independent of estrogen levels, are associated with the clinical manifestations in COVID-19 patients with high specificity.

## Introduction

The coronavirus infectious disease 2019 (COVID-19) pandemic, caused by severe acute respiratory syndrome coronavirus 2 (SARS-CoV-2) [1–3], is a huge challenge for public health throughout the world. Over 40 million cases and 1.1 million deaths have been reported globally as of 18 October 2020, with over 2.4 million new cases and 36000 new deaths reported over the past week [4]. SARS-CoV-2, which causes community-acquired pneumonia (CAP) with severe respiratory illness, is a member of the β coronaviruses, lineage B, and was first identified in Wuhan, China [5]. SARS-CoV-2, as many respiratory viruses, triggers the immune response of hosts and suppresses or even escapes the innate immune response, managing to establish infections and increase the efficiency of replication [6]. Patients with COVID-19 who develop from pneumonia to severe respiratory failure have hyper-inflammatory responses [7], which can be caused by either immune dysregulation or macrophage activation syndrome [8,9].

An increasing number of studies on immunopathology and biology have revealed the mechanics of viral infection and hyper-inflammatory responses [7,10]. Moreover, some clinical features of COVID-19 exhibit an association between patient sex and COVID-19 prevalence and severity; men are more predisposed to being affected by COVID-19 than women [11–15]. The immune response is a significant feature of sexual dimorphism, with women usually showing stronger immune responses. Sex differences in the immune response, especially the innate immune response, which have been implicated to influence infection outcomes in adults and children [16], are crucial in explaining the diversity of clinical features and for the efficient treatment of COVID-19 patients.

Lymphocyte and lymphocyte subset counts are of great value to ensure immune system functionality. Viral infections, immunodeficiency diseases, and other infectious diseases usually lead to abnormal changes in the numbers of lymphocyte subsets [17–19]. As a feature of the immune system, the CD4:CD8 ratio in the normal state is ∼2:1. But in some viral infections, this ratio is destroyed, and the CD4:CD8 ratio is inverted <1:1, indicating a serious immune disorder [20]. Studies have found that the CD4^+^ and CD8^+^ T cell counts are closely related to disease severity and clinical outcome, and therefore some authors proposed that CD4^+^ and CD8^+^ T cell counts in COVID-19 patients could be good biomarkers of COVID-19 activity [21,22]. The associations between (i) the differences in disease severity and prevalence between men and women and (ii) the differences in CD4^+^ and CD8^+^ T cell counts between male and female COVID-19 patients remain unclear. Therefore, investigations on the development of therapeutic and prophylactic approaches for COVID-19 need to include male–female differences [23].

The clinical features of COVID-19 are complicated and varied. In addition to lung injury, liver injury has been reported to occur during the course of the disease, and male patients, with significantly more males in the liver injury group than in the normal group [24]. C-reactive protein (CRP), aspartate aminotransferase (AST), alanine aminotransferase (ALT) as sensitive indicators of liver injury and inflammation can be used to compare the gender differences of nCOV patients. The Acute Physiology and Chronic Health Evaluation II (APACHE II) is the most commonly used severity-of-disease scoring system in ICUs around the world. Within the first 24 h of patient admittance, the worst value for each physiological variable is calculated into an integer score from 0 to 71. Higher scores represent a more severe disease and a higher hospital mortality risk [25]. APACHE II can be used to assess the severity of COVID-19 patients.

In the present work, we aim to clarify the characteristics and clinical significance of peripheral blood cell counts (BCCs) and the levels of CRP, AST, ALT, and APACHE II scores in male and female COVID-19 patients. Our results help to elucidate the pathogenesis of COVID-19 and to develop novel biomarkers and therapeutic strategies for male or severe COVID-19 cases.

## Materials and methods

### Study participants

The present study included a cohort of 111 people, i.e. 36 confirmed COVID-19 patients (the nCoV group, composed of 28 cases that were moderate and 8 cases that were severe on admission), 54 sex- and age-matched common viral CAP patients with respiratory symptoms (the VP group, composed influenza B 21 case, influenza A 15 case, respiratory syncytial virus 9 case, parainfluenza virus 7 case, and adenovirus 2 case), and 21 healthy controls (the HC group). Patients were enrolled between 31 January and 3 April 2020 at Xi’an Chest Hospital in Xi’an, Shannxi Province, China. COVID-19 and CAP patients with abnormalities in chest CT images were included. COVID-19 was confirmed by SARS-CoV-2 RNA real-time reverse transcription PCR, based on the 7th guidelines provided by the National Health Commission of China. Common viral CAP was confirmed by PNEUMOSLIDE IgM serological tests, and SARS-CoV-2 infection was excluded in these patients by nucleic acid and antibody tests. The HC group consisted of people who (i) were screened for COVID-19, (ii) had no respiratory symptoms, (iii) showed no lesions in chest CT images, and (iv) obtained negative results in SARS-CoV-2 nucleic acid and antibody tests.

The 36 cases of COVID-19 were divided into moderate (including moderate and mild) and severe (including severe and critically ill) groups. These diagnostic criteria were based on the recommendations by the Chinese National Institute for Viral Disease Control and Prevention. Moderate patients had symptoms like fever and respiratory tract symptoms, and imaging showed pneumonia. Severe patients had respiratory distress (respiratory rate ≥ 30 breaths/minute in a resting state) or a mean oxygen saturation of ≤93% (arterial blood oxygen partial pressure [PaO_2_]/oxygen concentration (FiO_2_) ≤ 300 mmHg). Written informed consents were signed by both patients and control members before participation. The study was approved by the Ethics Committees of Xi’an Chest Hospital.

### Data collection

Demographic features, clinical symptoms, and laboratory results were obtained from electronic medical records. Monocyte counts, lymphocyte subset counts, and CRP, AST, and ALT levels in the peripheral blood were measured at the time of early hospitalization (1–3 days after admission).

### Laboratory measurements

Real-time reverse-transcription PCR was performed using a SARS-CoV-2 nucleic acid detection kit following the manufacturer’s protocol (ShengXiang Bio-tech Co., Ltd., Changsha, China). Anti-SARS-CoV-2 IgM/IgG in serum samples were detected using colloidal-gold immunochromatography assay kits supplied by Lizhu Reagent Co., Ltd. (Zhuhai, China), following the manufacturer’s instructions. The PNEUMOSLIDE kit (Granada, Spain) was employed to detect IgM antibodies against nine common respiratory pathogens, i.e., *Legionella pneumophila* (pneumophila serogroup), *Mycoplasma pneumoniae* (*M. pneumoniae* in McCoy cells), *Coxiella burnetii* (*C. burnetii* in phase II), *Chlamydia pneumoniae* (*C. pneumoniae*, elementary bodies), adenovirus (Adenovirus in HEp-2 cells), respiratory syncytial virus (respiratory syncytial virus in HEp-2 cell), influenza A virus (Influenza A virus in LLC-MK2 cells), Influenza B virus (Influenza B virus in LLC-MK2 cells), and parainfluenza virus types 1, 2, and 3 (Parainfluenza virus types 1, 2, and 3 in LLC-MK2 cells). The detection was performed following the manufacturer’s instructions.

The proportions and counts of CD4^+^ T cells, CD8^+^ T cells, natural killer (NK) cells, and B cells were measured by flow cytometry. Antibodies against cell surface molecules were purchased from BD Company (Franklin Lakes, U.S.A.). Antibodies were used for flow cytometry are as follows: FITC anti-CD3 (SK7), PE anti-CD8 (SK1), PerCP anti-CD45 (HLe-1), SPC anti-CD4 (SK3), PE anti-CD16 (B73.1), PE anti-CD56 (NCAM 16.2), APC anti-CD19 (SJ25C1). All samples were detected and analyzed with a BD FACS Canto II Flow Cytometry System. Blood cells were detected and analyzed with a Mindray BC6800-plus automatic blood cell analyzer (Shenzhen, China). CRP, AST, and ALT levels were measured and analyzed with a Siemens 2400 automatic biochemical analyzer (Shanghai, China).

The APACHE II score was calculated based on 12 indicators including patient age, PaO_2_, temperature, mean arterial pressure, arterial pH, heart rate, respiratory rate, sodium, potassium, creatinine, hematocrit, white blood cell count, and Glasgow coma score. In addition, information about previous health conditions (history of surgery, history of organ insufficiency, and low immune function) is also calculated into the results.

### Statistical analysis

All data were entered in a pre-designed data collection form and checked to verify data accuracy. Qualitative data are presented as percentages and were compared by the Fisher’s exact test; quantitative data are presented as mean and interquartile range and were compared by the Mann–Whitney U test. Statistical significance was set at *P*<0.05 for two-sided tests. Correlations were analyzed by Spearman’s rank test. All statistical analyses were performed using SPSS, version 22.0 (IBM Corp., Armonk, NY).

## Results

### Clinical and laboratory baseline characteristics

**Figure 1:**
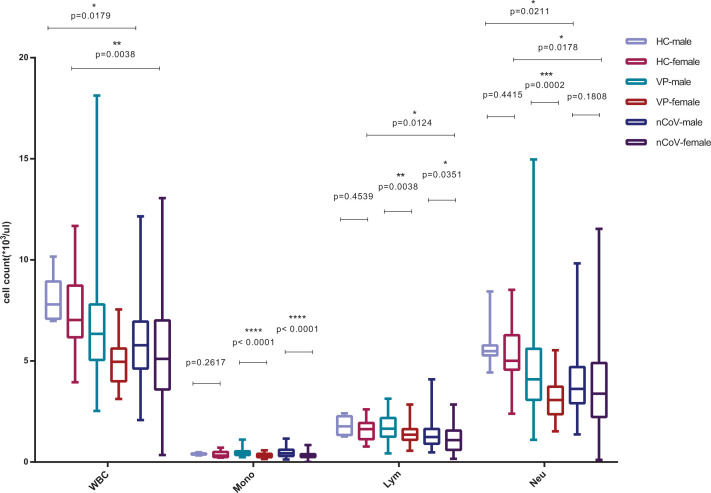
Characteristics of blood cells’ counts Blood cells’ counts, including white blood cells (WBC), monocyte (Mono), lymphocyte (Lym), and neutrophils (Neu) in the peripheral blood of healthy control (as HC) (male = 7, female = 14), common viral CAP cases (as VP) (male = 30, female = 24) and the COVID-19 patients (as nCoV) (male = 13, female = 23) classified into two categories by sex: male and female. Comparisons were done by the Mann–Whitney U test. *P<0.05; **P<0.01; ***P<0.001; ****P<0.0001.

**Figure 2:**
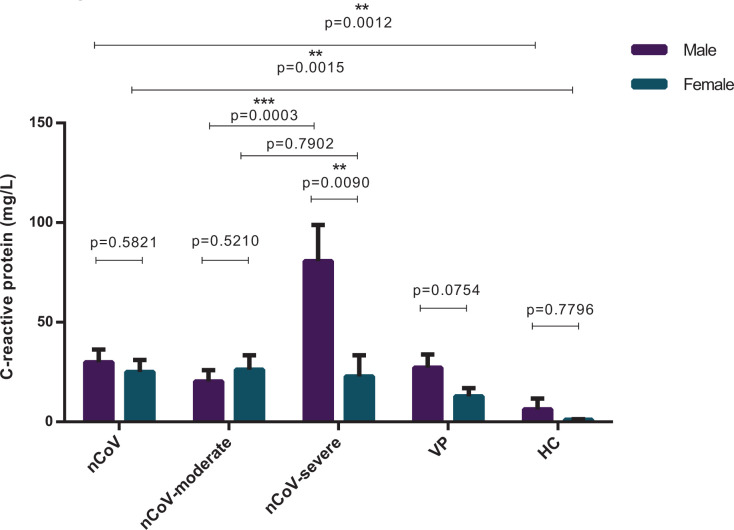
The levels of CRP The levels of CRP of COVID-19 patients classified further according to disease severity on admission, as moderate (nCoV-moderate) (male = 10, female = 18) and severe (nCoV-severe) (male = 3, female = 5) comparing with healthy crew (as HC) (male = 7, female = 14) and common viral CAP cases (as VP) (male = 30, female = 24). Each bar and error bar represents mean value and standard error. Comparisons were done by the Mann–Whitney U test. **P<0.01; ***P<0.001.

The present study included a cohort of 111 people, i.e. 36 confirmed COVID-19 patients (the nCoV group), including 28 moderate and 8 severe cases on admission, 54 common viral CAP patients with respiratory symptoms (the VP group), and 21 healthy controls (the HC group). Baseline clinical and laboratory characteristics of the cohort are presented in Table 1. No significant differences with respect to age were found between males and females within each group or between groups, except that the age of females in the nCoV group (52.4 years) was higher than that of females in the HC group (35.6 years, *P*=0.0037). Cough (58.3%) and fever (77.8%) were the main symptoms of COVID-19 patients. SARS-CoV-2 and other common virus infection routes showed the same prevalence between males and females, and no significant differences were found with respect to comorbidities between males/females in nCoV group and VP group. In female COVID-19 patients, the disease was less severe than in males on the basis of the APACHE II (*P*=0.0296) [25]. The laboratory values of BCCs and CRP levels in the peripheral blood demonstrated lower white blood cell, lymphocyte, and neutrophil counts and higher CRP in both VP and nCoV groups than HC group, monocyte count higher in males and lower in females in both VP and nCoV group than HC groups; higher white blood cell (*P*=0.0001), higher lymphocyte (*P*=0.0002), higher neutrophil counts (*P*=0.0038), higher monocyte count (*P*<0.0001) was found in male than in female common viral CAP patients (Table 1, Figure 1). And higher lymphocyte (*P*=0.0351), higher monocyte count (*P*<0.0001), higher ALT (*P*=0.0068) was found in male than in female COVID-19 patients (Table 1, Figure 1). However, higher CRP (*P*=0.0090) were found in male than in female severe COVID-19 patients (Figure 2), whereas these differences were non-significant between males and females in moderate COVID-19 patients, common viral CAP patients and healthy person.

**Table 1:** Baseline clinical and laboratory characteristics of the cohorts of viral CAP and of pneumonia caused by SARS-CoV-2

	Healthy crew (*n*=21)			Viral (*n*=54)			SARS-CoV-2 (*n*=36)		
	Male (*n*=7)	Female (*n*=14)	*P*-value	Male (*n*=30)	Female (*n*=24)	*P*-value	Male (*n*=13)	Female (*n*=23)	*P*-value
**Characteristics**									
Age (years, mean ± SD)	38.1 (31.0–45.3)	35.6 (29.6–41.7)	0.5859	45.7 (40.6–50.8)	43.4 (35.9–50.9)	0.5962	49.2 (38.6–59.9)	52.4 (44.9–59.8)	0.6079
**Epidemiology, number (%)**						0.6899			0.4868
Close contact exposure	-	-	-	23.3	33.3	-	61.5	73.9	-
Travel history	-	-	-	6.67	4.17	-	23.1	8.70	-
Uncertain	-	-	-	70.0	62.5	-	15.4	17.4	-
**Comorbidities, number (%)**									
Diabetes	-	-	-	3.3	0.00	1.000	7.7	0.00	0.3611
Hypertension	-	-	-	10.0	4.2	0.6204	15.4	13.0	1.000
Heart diseases	-	-	-	3.3	0.00	1.000	0.00	4.3	1.000
**Symptoms, number (%)**									
Fever	-	-	-	73.3	79.2	0.7527	84.6	73.9	0.6820
Cough	-	-	-	70.0	66.7	1.000	53.8	60.9	0.7356
Chest tightness	-	-	-	16.7	8.3	0.4432	7.7	26.1	0.3822
Diarrhea	-	-	-	0.00	0.00	1.000	23.1	13.0	0.6454
Fatigue	-	-	-	10.0	12.5	1.000	15.4	34.8	0.2698
**APACHE II score**	-	-	-	6.41 (5.12–7.70)	5.06 (3.45–6.67)	0.1943	8.50 (4.63–7.59)	6.11 (7.16–9.84)	0.0296^1^
**Laboratory values**									
WBC count (*10^3^/μl)	8.07 (6.99–9.14)	7.49 (6.16–8.81)	0.2178	6.78 (6.10–7.46)	4.93 (4.62–5.24)	0.000^4^	6.01 (5.42–6.60)	5.33 (4.77–5.90)	0.0771
Neutro count (*10^3^/μl)	5.77 (4.61–6.93)	5.47 (4.45–6.48)	0.4415	4.41 (3.79–5.03)	3.12 (2.88–3.36)	0.0002^3^	4.07 (3.57–4.57)	3.85 (3.31–4.38)	0.1808
Lympho count *10^3^/μl)	1.79 (1.35–2.24)	1.59 (1.27–1.91)	0.4539	1.71 (1.54–1.88)	1.36 (1.24–1.49)	0.0038^2^	1.38 (1.20–1.56)	1.13 (1.00–1.27)	0.0351^1^
Monocyte count (*10^3^/μl)	0.40 (0.36–0.45)	0.37 (0.28–0.45)	0.2617	0.48 (0.43–0.53)	0.33 (0.30–0.36)	0.000^4^	0.51 (0.43–0.58)	0.32 (0.28–0.36)	0.000^4^
PLT count (*10^3^/μl)	276 (241–310)	299 (255–345)	0.4572	230 (216–244)	255 (241–269)	0.0115^1^	237 (208–265)	233 (206–260)	0.8668
ALT (U/l)	-	-	-	35.9 (30.2–41.5)	–38.5 (29.9–47.0)	0.1784	71.7 (51.7–91.7)	43.4 (32.9–53.9)	0.0068^2^
AST (U/l)	-	-	-	22.8 (19.7–25.9)	26.0 (19.4–32.7)	0.2103	34.9 (23.2–46.6)	27.8 (22.2–33.5)	0.1184
CRP (U/l)	0.93 (0.00–1.89)	1.06 (0.50–1.62)	0.4856	27.2 (13.8–40.7)	12.8 (4.52–21.1)	0.0754	29.9 (16.9–42.9)	25.1 (13.2–37.0)	0.5821

Comparisons within the respective groups by the Mann–Whitney U test: ^1^*P*<0.05; ^2^*P*<0.01; ^3^*P*<0.001; ^4^*P*<0.0001. Abbreviations: Lympho, lymphocyte; Neutro, neutrophil; PLT, platelet.

### Circulating monocyte counts, CD4^+^ T cell and CD8^+^ T cell proportions, and the CD4^+^/CD8^+^ ratio were different between male and female COVID-19 patients, independent of estrogen level

**Figure 3:**
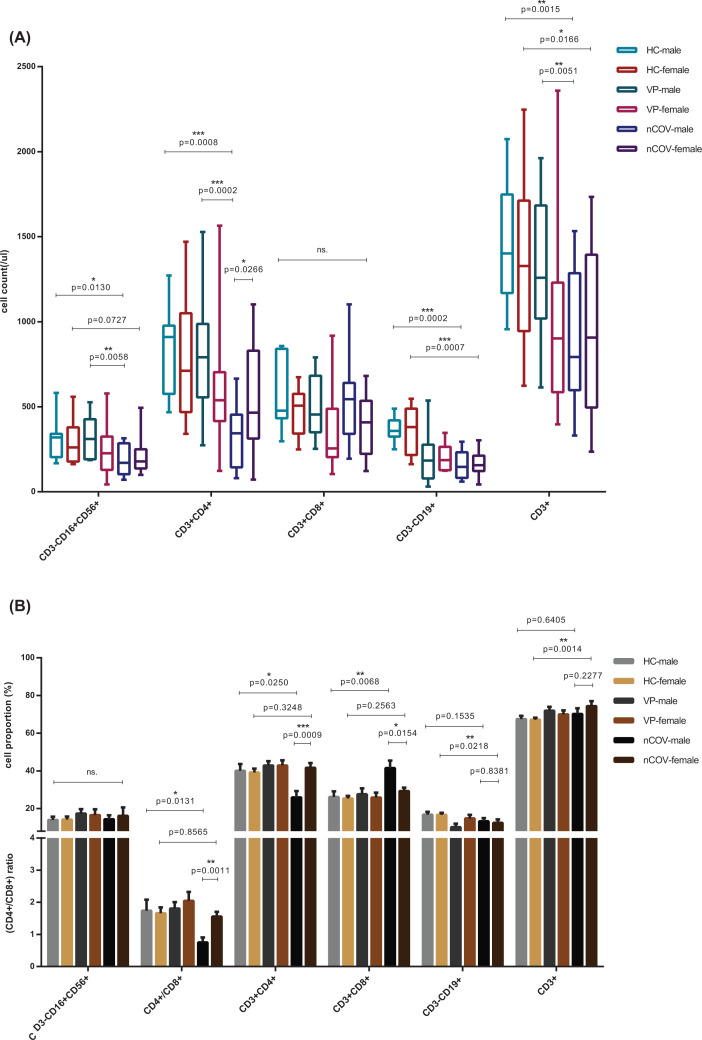
Features of lymphocyte subset Cell counts (**A**) and cell proportions (**B**) of lymphocyte subset, consist of CD3^−^CD16^+^CD56^+^, CD3^+^CD4^+^, CD3^+^CD8^+^, CD3^−^CD19^+^ and CD3^+^,which represents NK cell, CD4^+^T cell, CD8^+^T cell, total B lymphocyte and total T lymphocyte, respectively, of healthy control (as HC) (male = 7, female = 14), common viral CAP cases (as VP) (male = 30, female = 24) and the COVID-19 patients (as nCoV) (male = 13, female = 23) classified into two categories by sex: male and female. Statistical comparisons are indicated by the arrows; ns., non-significant; Comparisons were done by the Mann–Whitney U test. *P<0.05; **P<0.01; ***P<0.001; ****P<0.0001.

The lymphocyte subset counts, including NK (CD3^−^CD16^+^CD56^+^) cells, T (CD3^+^) cells, and B (CD3^−^CD19^+^) cells, were analyzed, as these cells reflect the function and balance of the immune system while the cell proportions partially represent the degree of active cell proliferation (Figure 3). Lymphopenia was common in respiratory viral CAP patients, but more severe in COVID-19 patients (Figure 3A, Supplementary Table S1). Abnormal proportions of CD3^+^CD4^+^ T cells and CD3^+^CD8^+^ T cells, resulting in an anomalous CD4^+^/CD8^+^ ratio, indicated the immune system was dysregulated. Significant differences were observed between COVID-19 and common viral CAP patients (Figure 3A,B) and between male and female COVID-19 patients (Figure 3A,B). Male COVID-19 patients experienced more severe immune dysregulation, with lower CD4^+^ T cell proportions (*P*=0.0250), higher CD8^+^ T cell proportions (*P*=0.0068), and much lower CD4^+^/CD8^+^ ratios (*P*=0.0131), indicating a possible immune deficiency that is similar to that observed in HIV infections [26] (Figure 3B, Supplementary Table S2). No significant differences were found with respect to the proportions of lymphocyte subsets between healthy and COVID-19 females, except for lower total B cell and higher total T cell proportions in the latter.

**Figure 4:**
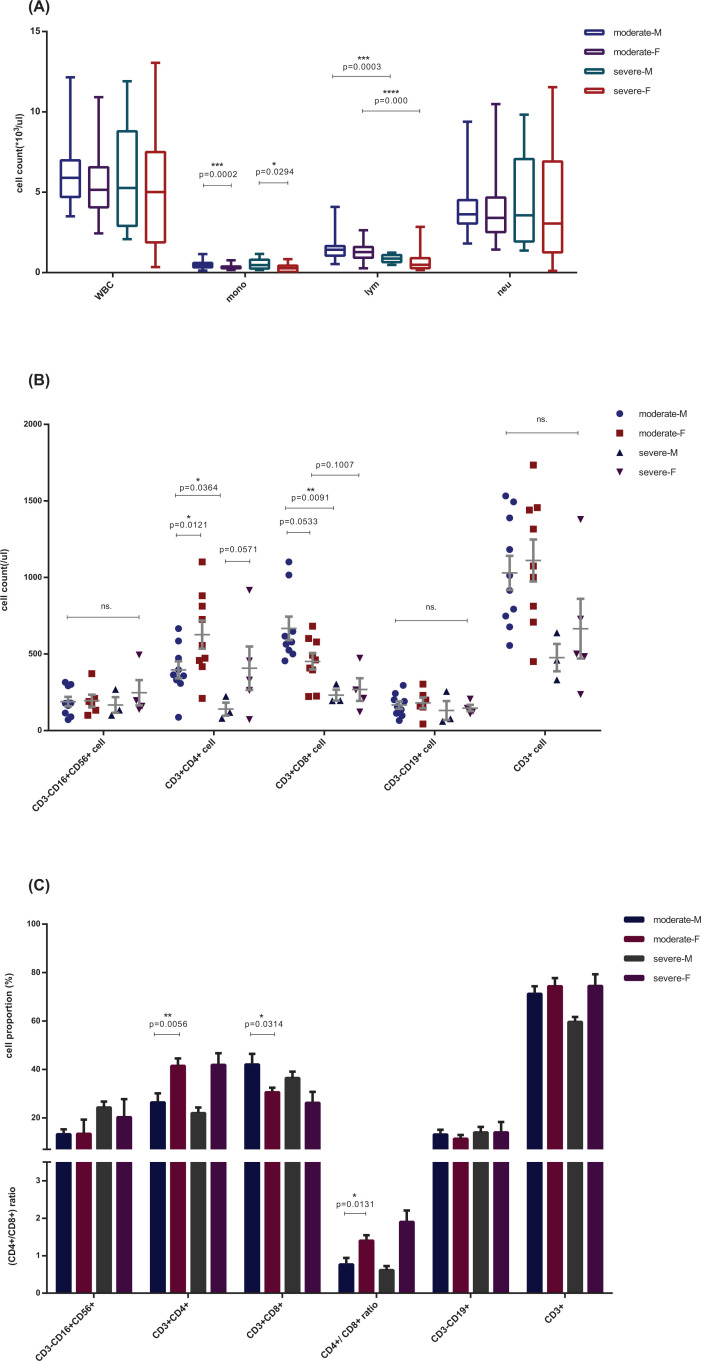
Comparisons of immune-related indices in COVID-19 patients classified by disease severity and sex Cell counts of whole blood cell (**A**) and lymphocyte subset (**B**) and the cell proportions of lymphocyte subset, consist of CD3-CD16^+^CD56^+^, CD3^+^CD4^+^, CD3^+^CD8^+^, CD3-CD19^+^ and CD3^+^,which represents NK cell, CD4^+^ T cell, CD8^+^ T cell, total B lymphocyte and total T lymphocyte, respectively (**C**) of patients with COVID-19, classified into four groups according to the disease severity, moderate (male = 10, female = 18) and severe (male = 3, female = 5), and patient sex, male (M) and female (F). Comparisons were done by the Mann–Whitney U test. *P<0.05; **P<0.01; ***P<0.001; ****P<0.0001.

**Figure 5:**
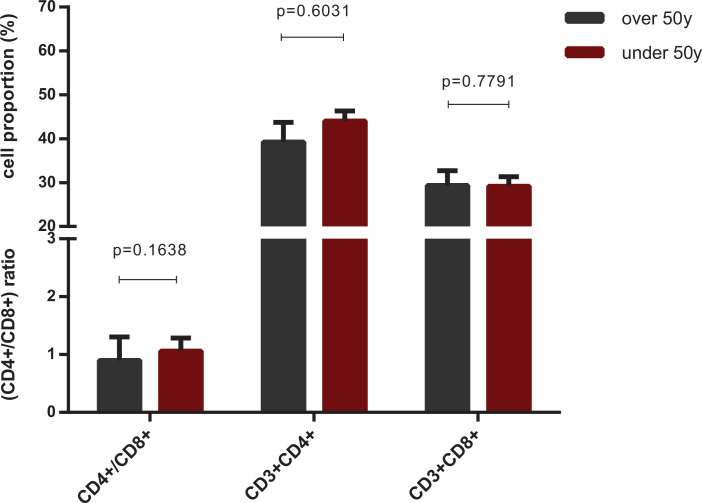
Cell proportions of lymphocyte subset in female COVID-19 patients with different levels of estrogen The cell proportions of lymphocyte subset of COVID-19 female cases, classified into two age groups: under (38 years, 31–44) (*n*=9) and over 50 (68 years, 60–76) (*n*=14) (*P*<0.0001) years old, which is the average age of women’s menopause with much lower level of estrogen. Non-significant differences in CD4^+^ T, CD8^+^ T and the ratio of CD4^+^/CD8^+^, which was the characteristics of infection by SARS-CoV-2 and associated with the disease severity, between the two groups demonstrated that the differentiations of immune response may be independent of the level of estrogen. Comparisons were done by the Mann–Whitney U test.

The difference in immune dysregulation, with a lower CD4^+^ T cell proportion, a higher CD8^+^ T proportion, and higher monocyte counts, observed in patients of different sex showed a similar pattern between COVID-19 patients with distinct disease severity, which was of stronger statistical significance (Figure 4). The difference was most clear when COVID-19 patients were classified on the basis of both sex and disease severity. CRP levels were much higher in severe male cases than in severe female cases (*P*=0.0090), whereas no significant difference was observed between moderate and severe females (*P*=0.5210) (Figure 2). BCCs showed lower lymphocyte counts in the severe group, in both male (*P*<0.0001) and female patients (*P*=0.0003), and different monocyte counts between men and women, in both moderate (*P*=0.0002) and severe (*P*=0.0294) cases (Figure 4A, Supplementary Table S3). Lower CD4^+^ T cell counts (*P*=0.0121), higher CD8^+^ T cell counts (*P*=0.0533), and an abnormal CD4^+^/CD8^+^ ratio (*P*=0.0131) were observed in men of the moderate COVID-19 group, and severe symptoms were associated with lower CD4^+^ and CD8^+^ T cell proportions in all patients, with much lower proportions in severe males (Figure 4B,C and Supplementary Tables S4 and S5). This difference was independent of female estrogen levels, as indicated by the fact that we did not observe a significant difference in the biomarkers of immune dysregulation between female patients aged below (38 ± 6.44 years) and above (67.78 ± 7.84 years) 50 years (Figure 5), which is the average age of menopause, after which estrogen levels drop substantially [27,28]. The representative flow cytometry charts of severe COVID-19 patients, moderate COVID-19 patients, common viral CAP cases, and healthy control in both male and female are depicted in Supplementary Figure S1.

### The elevated CD8^+^ T cell proportion is associated with a decreased CD4^+^ T cell proportion in male, but not female, COVID-19 patients

**Figure 6:**
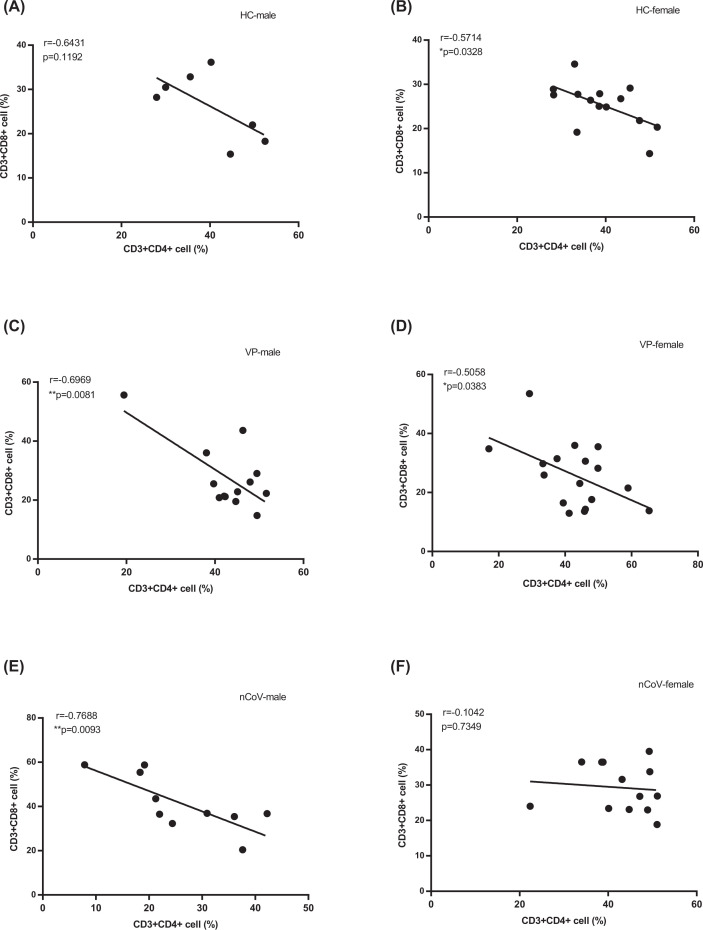
Correlations between proportions of CD4^+^ and CD8^+^ T cells Correlations between proportions of CD4^+^ T cell and CD8^+^ T cell in male and female of healthy crew (HC male (**A**) (*n*=7) and HC female (**B**) (*n*=14)), common viral CAP patients (VP-male (**C**) (*n*=30) and VP-female (**D**) (*n*=24) and the COVID-19 cases (nCoV-male (**E**) (*n*=13) and nCoV-female (**F**) (*n*=23)). *P*-values (two-sided) and r values were based on Spearman’s rank test.

The potential correlation between CD4^+^ T cell proportion and the proportion of CD8^+^ T cells or monocytes in the entire cohort was analyzed by Spearman rank-order correlation (Figure 6). As demonstrated in Figure 6, the proportion of CD4^+^ T cells was negatively correlated with the proportion of CD8^+^ T cells in male COVID-19 patients (Figure 6E; r = −0.7688, *P*=0.0093), as well as in males and females in the VP (Figure 6C,D) and HC groups (Figure 6A,B), whereas no significant correlation was observed in female COVID-19 patients (Figure 6F; r = −0.1042, *P*=0.7349). No associations with counts and proportions of other lymphocytes and monocytes were found.

## Discussion

The COVID-19 pandemic endangers global health. Studies have reported many differences between male and female COVID-19 patients in prevalence [11], disease severity, and mortality [12,13]; higher incidence, susceptibility, and mortality rates have been reported in men in several countries, and therefore the male sex is considered as a poor prognostic factor [11,29], as men are more susceptible [14,15] than women to viral infections, in particular to SARS-CoV-2, due to differences in innate immunity, steroid hormone levels, and factors related to sex chromosomes [16,30,31]. In the present study, a higher APACHE II score, which is a measure for acute physiology and chronic health [25], higher monocyte counts, and higher CRP and ALT levels were found in male COVID-19 patients than in their age- and severity-matched female counterparts, confirming the difference in disease severity and innate immune response between male and female COVID-19 patients.

Moreover, studies on the immunopathology have suggested that the development of COVID-19 to severe respiratory failure is driven by a unique pattern of immune dysregulation, with two key features: overproduction of pro-inflammatory cytokines by monocytes and dysregulation of lymphocytes, characterized by CD4 lymphopenia and subsequently B cell lymphopenia [8]. Similarly, we found lower CD4^+^ T proportions and higher monocyte counts in males, and both CD4 and CD8 lymphopenia in patients with severe COVID-19 of both sexes, whereas an elevated CD8^+^ T proportion was commonly found in moderate patients, with males exhibiting higher CD8^+^ T cell proportions than females. This implies that stronger immune dysregulation, which is associated with poor outcomes in SARS-CoV-2-infected individuals, was observed in male than in female COVID-19 patients.

Studies have shown that female patients mounted significantly more robust T cell activation than male patients during SARS-CoV-2 infection, which was sustained in old age. They also found that a poor T cell response negatively correlated with patients’ age and was associated with worse disease outcome in male patients, but not in female patients. Conversely, higher innate immune cytokines in female patients associated with worse disease progression, but not in male patients [32].

Lephart et al. think the average age of menopause in women is 50, after which estrogen levels drop dramatically [27]. By the fact that we did not observe a significant difference in the biomarkers of immune dysregulation between female patients aged below (38 ± 6.44 years) and above (67.78 ± 7.84 years) 50 years, we hypothesized that immune dysregulation was independent of estrogen level. Of course, there are other age-related factors that need to be further explored. Previous studies on SARS-CoV, which is closely related to SARS-CoV-2, have shown an increased susceptibility in male mice, which is associated with high virus titers, increased vascular leakage, and alveolar edema, which is accompanied by higher accumulation of inflammatory monocytes/macrophages and neutrophils in the lungs [33]. Furthermore, sex-specific differences, independent of T and B cell responses [30], and a protective effect of the estrogen receptor signaling pathway were reported in SARS-CoV-infected mice [33]. Our present work shows that decreased lymphocyte subset counts and proportions are common in COVID-19 patients, except for the CD3^+^ and CD8^+^ T cell proportions. Lower CD4^+^ T cell proportions, increased CD8^+^ T cell proportions, the consequent abnormal CD4^+^/CD8^+^ ratio, and higher monocyte counts were significantly associated with the male sex and disease severity, independently of estrogen levels, indicating the sex-specific differences may be related to sex chromosomes, even though in women only one X chromosome, encoding the immune regulatory genes, is active, causing lower viral loads and less inflammation than in men, as higher CD4^+^ T cell counts and proportions are associated with a better immune response.

Deeks et al. and others reported an immunologic activation ‘set point,’ which varies widely between individuals but is generally stable in the same ones, is established in early viral infections, and the CD8^+^ T cell activation set point is a strong independent predictor of the rate of CD4^+^ T cell decline [34]. Studies have shown that there was a firm correlation between the highest values of inflammation indicators with the decrease in percentage of CD8 T lymphocytes in COVID-19 [35]. Wang et al. found that compared with patients with mild illness, severe cases had significantly lower total lymphocytes, CD4^+^ T cells, CD8^+^ T cells, and B cells. No significant difference was observed in CD4^+^/CD8^+^ ratio and NK cells. Our study showed lower lymphocyte counts in the severe group, in both male (*P*<0.0001) and female patients (*P*=0.0003). But our study found that compared with female patients, the difference of CD4^+^ T cells and CD8^+^ T cells between male patients in severe nCoV group and moderate nCoV group was more significant, which suggests that there may be a different immune response between men and women. Interestingly, the correlation between CD4^+^ T and CD8^+^ T proportions showed a negative association in all three groups (nCoV, VP, and HC) except that non-significant correlation was found in female COVID-19 patients, for unclear reasons. It may be related to a female-specific immune response to SARS-CoV-2 infection, but this hypothesis requires further investigations on the differences in cytokine levels and expression levels of immune-related genes between men and women. In addition, corticosteroid therapy significantly significant increase in total lymphocytes, CD8^+^ T cells, and B cells in responsive cases. Therefore, corticosteroid therapy has become a potential treatment for patients infected with the SARS-CoV-2 [36].

Previous studies on the lymphocyte subset counts showed close relations between (i) CD4^+^ and CD8^+^ T cell counts and (ii) disease severity and clinical outcome, and suggested that the CD4^+^ and CD8^+^ T cell counts in patients with COVID-19 could be good biomarkers of COVID-19 activity [21,22]. In the present study, we obtained similar results in male patients, but quite different results in female patients, suggesting that sex-dependent, rather than unisex, biomarkers should be used to predict the severity and prognosis of COVID-19 patients.

We wish to highlight several limitations of the present study. Firstly, our sample size was limited, which could reduce the robustness of our conclusions. Secondly, we did not measure the serum levels of cytokines. Measurements of the levels of pro-inflammatory cytokines, which are secreted by monocytes, macrophages, and lymphocytes, will improve our understanding of the mechanisms underlying the differences between male and female COVID-19 patients with respect to the changes in their immune responses upon SARS-CoV-2 infection, rather than only identifying associations. Finally, in the present study we did not measure the expression levels of immune-related genes, which could tell us more about the factors influencing the immune response against SARS-CoV-2 and aid in the development of an effective treatment for male and severe COVID-19 patients.

We have demonstrated that immune dysregulation is more severe in male than in female COVID-19 patients, which may be related to the sex-related differences in the immune activation ‘set point,’ resulting in different disease severity and mortality between men and women. Furthermore, due to such difference between different sexes in biomarkers and laboratory values, which may be used to evaluate the severity or prognosis, sex-dependent diagnosis and treatment methods may be needed for accurate and effective therapy.

## Perspectives

- Investigation of sex-specific differences in clinical features and laboratory indices of COVID-19, which are consistent with immune responses to SARS-CoV-2 and immune dysregulation related, compared with sex- and age-matched common viral CAP patients and 21 healthy controls.
- Providing more evidence that the sex-specific differences observed in the present study, independent of estrogen level, are associated with COVID-19 prevalence and severity differed between men and women with high specificity.
- We emphasize that sex-dependent diagnostic and treatment methods may be needed for accurate and effective therapy considering sex as a biological variable in COVID-19.

## Supplementary Material

Supplementary Figure S1 and Tables S1-S5

## Data Availability

All data used during the study are available from the corresponding author on request.

## Abbreviations

ALT: alanine aminotransferase
APACHE II: Acute Physiology and Chronic Health Evaluation II
AST: aspartate aminotransferase
BCC: peripheral blood cell count
CAP: community-acquired pneumonia
COVID-19: coronavirus infectious disease 2019
CRP: C-reactive protein
NK: natural killer
PaO_2_: oxygen partial pressure
SARS-CoV-2: severe acute respiratory syndrome coronavirus 2
