# Supplementary material for: Sex differences in immune responses to SARS-CoV-2 in patients with COVID-19
Source: Biosci Rep. 2021 Jan 29;41(1):BSR20202074. doi: 10.1042/BSR20202074 (PMC7846967; doi:10.1042/BSR20202074)
Supplement: Supplementary Figure S1 and Tables S1-S5 [file BSR-2020-2074_supp.pdf]

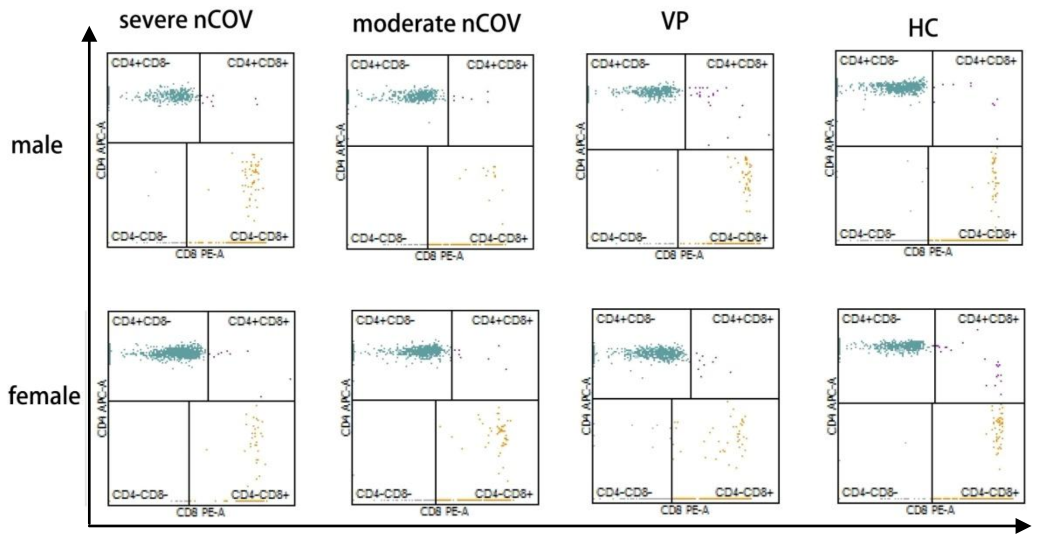


Figure S1. The representative flow cytometry charts.

The representative flow cytometry charts of severe COVID-19 patients (as severe nCOV), moderate COVID-19 patients (as moderate nCOV), common viral community-acquired pneumonia cases (as VP) and healthy control (as HC) in both male and female.

Table S1 The representative comparison of lymphocyte subset cell counts between HC,VP and COVID-19 patients followed by the mean, SD and p-value .

| | | Mean±SD | P value |
|---|---|---|---|
| *NK cell* *（$CD3^-CD16^+CD56^+$）* | HC-female vs. | 294.43(167.33-421.52) | 0.7942 |
| | HC-male | 318.29(184.50-452.07) | |
| | VP-female vs. | 248.00(85.37-410.63) | 0.3724 |
| | VP-male | 315.00(189.14-440.86) | |
| | nCoV-female vs. | 216.20(93.95-338.45) | 0.4824 |
| | nCoV-male | 184.00(95.47-272.53) | |
| | nCoV-male vs. | 184.00(95.47-272.53) | 0.0130* |
| | HC-male | 318.29(184.50-452.07) | |
| | nCoV-female vs. | 216.20(93.95-338.45) | 0.0727 |
| | HC-female | 294.43(167.33-421.52) | |
| | VP-male vs. | 315.00(189.14-440.86) | 0.9305 |
| | HC-male | 318.29(184.50-452.07) | |
| | VP-female vs. | 248.00(85.37-410.63) | 0.4656 |
| | HC-female | 294.43(167.33-421.52) | |
| *Th cell($CD3^+CD4^+$）* | HC-female vs. | 776.14(405.21-1147.08) | 0.5331 |
| | HC-male | 858.86(591.33-1126.38) | |
| | VP-female vs. | 632.47(275.66-989.28) | 0.0783 |
| | VP-male | 802.31(482.85-1121.77) | |
| | nCoV-female vs. | 548.21(248.88-847.55) | 0.0266* |
| | nCoV-male | 332.50(145.87-519.13) | |
| | nCoV-male vs. | 332.50(145.87-519.13) | 0.0008*** |
| | HC-male | 858.86(591.33-1126.38) | |
| | nCoV-female vs. | 548.21(248.88-847.55) | 0.1529 |
| | HC-female | 776.14(405.21-1147.08) | |
| | VP-female vs. | 632.47(275.66-989.28) | 0.3741 |
| | HC-female | 776.14(405.21-1147.08) | |
| | VP-male vs. | 802.31(482.85-1121.77) | 0.4833 |
| | HC-male | 858.86(591.33-1126.38) | |
| | nCoV-male vs. | 332.50(145.87-519.13) | 0.0002*** |
| | VP-male | 802.31(482.85-1121.77) | |
| *Ts cell($CD3^+CD8^+$)* | HC-female vs. | 467.64(333.68-601.60) | 0.582 |
| | HC-male | 560.86(346.76-774.96) | |
| | VP-female vs. | 387.59(116.11-659.07) | 0.0859 |
| | VP-male | 490.00(302.81-677.19) | |
| | nCoV-female vs. | 395.38(221.44-569.33) | 0.091 |
| | nCoV-male | 558.30(278.59-838.01) | |
| | nCoV-male vs. | 558.30(278.59-838.01) | 0.8666 |
| | HC-male | 560.86(346.76-774.96) | |
| | nCoV-female vs. | 395.38(221.44-569.33) | 0.2358 |
| | HC-female | 467.64(333.68-601.60) | |
| *B cell($CD3^-CD19^+$)* | nCoV-male vs. | 157.70(78.43-236.97) | 0.618 |
| | VP-male | 206.13(46.15-366.10) | |
| | nCoV-female vs. | 167.70(96.27-239.13) | 0.0007*** |
| | HC-female | 363.86(218.31-509.41) | |
| | nCoV-female vs. | 167.70(96.27-239.13) | 0.5647 |
| | VP-female | 203.75(118.74-288.76) | |

| | | | |
|---|---|---|---|
| | HC-male vs. | 373.86(296.70-451.02) | 0.0002*** |
| | nCoV-male | 157.70(78.43-236.97) | |
| *T cell($CD3^+$)* | nCoV-male vs. | 902.38(507.25-1297.52) | 0.0015** |
| | HC-male | 1472.86(1136.77-1808.95) | |
| | nCoV-female vs. | 951.64(491.50-1411.79) | 0.0166* |
| | HC-female | 1371.64(879.88-1863.40) | |
| | VP-female vs. | 986.13(493.37-1478.89) | 0.0038** |
| | VP-male | 1342.86(941.02-1744.70) | |
| | nCoV-female vs. | 951.64(491.50-1411.79) | 0.8915 |
| | nCoV-male | 902.38(507.25-1297.52) | |
| | nCoV-male vs. | 902.38(507.25-1297.52) | 0.0051** |
| | VP-male | 1342.86(941.02-1744.70) | |
| | nCoV-female vs. | 951.64(491.50-1411.79) | > 0.9999 |
| | VP-female | 986.13(493.37-1478.89) | |

Cell counts of lymphocyte subset, consist of NK cell（$CD3\text{-}CD16^+CD56^+$）, Th cell($CD3^+CD4^+$）, Ts cell($CD3^+CD8^+$), total B lymphocyte($CD3^-CD19^+$) and total T lymphocyte($CD3^+$), respectively, of healthy control (as HC)(male=7,female=14), common viral community-acquired pneumonia cases (as VP)(male=30,female=24) and the COVID-19 patients (as nCoV)(male=13,female=23) classified into two categories by sex: male and female. Statistical comparisons are indicated by the arrows; ns.: non-significant; *$p < 0.05$; **$p < 0.01$; ***$p < 0.001$; ****$p < 0.0001$. Comparisons were done by the Mann-Whitney U test.

Table S2 The representative comparison of lymphocyte subset cell proportions between HC,VP and COVID-19 patients followed by the mean, SD and p-value

| | Mean±SD | P value | |
|---|---|---|---|
| *NK cell($CD3^-CD16^+CD65^+$)* | nCoV-male vs. | 14.34(12.21 16.47) | 0.7298 |
| | HC-male | 13.94(12.18 15.71) | |
| | nCoV-female vs. | 16.17(11.67 20.67) | 0.4279 |
| | HC-female | 14.19(12.64 15.75) | |
| | nCoV-male vs. | 14.34(12.21 16.47) | 0.3127 |
| | VP-male | 17.33(14.95-19.71) | |
| | nCoV-female vs. | 16.17(11.67 20.67) | 0.5647 |
| | VP-female | 16.52(13.37-19.67) | |
| *Th cell($CD3^+CD4^+$)* | nCoV-male vs. | 0.75(0.59 0.91) | 0.0131* |
| */ Ts cell($CD3^+CD8^+$)* | HC-male | 1.74(1.40 2.08) | |
| | nCoV-female vs. | 1.56(1.41 1.70) | 0.8565 |
| | HC-female | 1.66(1.47 1.84) | |
| | nCoV-female vs. | 1.56(1.41 1.70) | 0.0011** |
| | nCoV-male | 0.75(0.59 0.91) | |
| *Th cell($CD3^+CD4^+$)* | nCoV-male vs. | 25.98(22.65 29.32) | 0.0250* |
| | HC-male | 40.06(36.49 43.62) | |
| | nCoV-female vs. | 41.64(39.14 44.15) | 0.3248 |
| | HC-female | 39.19(37.16 41.22) | |
| | nCoV-female vs. | 41.64(39.14 44.15) | 0.0009*** |
| | nCoV-male | 25.98(22.65 29.32) | |
| *Ts cell($CD3^+CD8^+$)* | nCoV-male vs. | 41.48(37.50 45.47) | 0.0068** |
| | HC-male | 26.19(23.25 29.13) | |
| | nCoV-female vs. | 29.26(27.39 31.13) | 0.2563 |
| | HC-female | 25.33(23.99 26.68) | |
| | nCoV-female vs. | 29.26(27.39 31.13) | 0.0154* |
| | nCoV-male | 41.48(37.50 45.47) | |
| *B cell($CD3^-CD19^+$)* | nCoV-male vs. | 13.24(11.50 14.98) | 0.1535 |
| | HC-male | 16.80(15.31 18.29) | |
| | nCoV-female vs. | 12.42(10.56 14.28) | 0.0218* |
| | HC-female | 16.66(15.55 17.77) | |
| | nCoV-female vs. | 12.42(10.56 14.28) | 0.8381 |
| | nCoV-male | 13.24(11.50 14.98) | |
| *T cell($CD3^+$)* | nCoV-male vs. | 70.17(67.14 73.21) | 0.6405 |
| | HC-male | 67.51(65.77 69.25) | |
| | nCoV-female vs. | 74.38(71.71 77.06) | 0.0014** |
| | HC-female | 67.15(66.05 68.24) | |
| | nCoV-female vs. | 74.38(71.71 77.06) | 0.2277 |
| | nCoV-male | 70.17(67.14 73.21) | |

Cell proportions lymphocyte subset, consist of NK cell（$CD3^-CD16^+CD56^+$）, Th cell($CD3^+CD4^+$）, Ts cell($CD3^+CD8^+$), total B lymphocyte($CD3^-CD19^+$) and total T lymphocyte($CD3^+$), respectively, of healthy control (as HC)(male=7,female=14), common viral community-acquired pneumonia cases (as VP)(male=30,female=24) and the COVID-19 patients (as nCoV)(male=13,female=23) classified into two categories by sex: male and female. Statistical comparisons are indicated by the arrows; ns.: non-significant; *$p < 0.05$; **$p < 0.01$; ***$p < 0.001$; ****$p < 0.0001$. Comparisons were done by the Mann-Whitney U test.

Table S3 The representative comparison of cell counts of whole blood cell in COVID-19 patients classified by disease severity and sex followed by the mean, SD and p-value

| | | Mean±SD | P value |
|---|---|---|---|
| *mono* | moderate-F vs. | 0.34 (0.23-0.45) | 0.0002*** |
| | moderate-M | 0.50(0.24-0.77) | |
| | severe-F vs. | 0.29(0.05-0.52) | 0.0294* |
| | severe-M | 0.52(0.17-0.86) | |
| | severe-M vs. | 0.52(0.17-0.86) | 0.7154 |
| | moderate-M | 0.50(0.24-0.77) | |
| | severe-F vs. | 0.29(0.05-0.52) | 0.2911 |
| | moderate-F | 0.34 (0.23-0.45) | |
| *lym* | moderate-F vs. | 1.30 (0.79-1.82) | 0.2305 |
| | moderate-M | 1.48(0.82-2.15) | |
| | severe-F vs. | 0.78(0.08-1.48) | 0.0843 |
| | severe-M | 0.87(0.61-1.13) | |
| | severe-M vs. | 0.87(0.61-1.13) | 0.0003*** |
| | moderate-M | 1.48(0.82-2.15) | |
| | severe-F vs. | 0.78(0.08-1.48) | < 0.0001**** |
| | moderate-F | 1.30 (0.79-1.82) | |

Cell counts of whole blood cell of patients with COVID-19, classified into 4 groups according to the disease severity, moderate (male=10,female=18) and severe (male=3,female=5), and patient sex, male (M) and female (F). Comparisons were done by the Mann-Whitney U test.

Table S4 The representative comparison of cell counts of lymphocyte subset in COVID-19 patients classified by disease severity and sex followed by the mean, SD and p-value

| | | *Mean ±SD* | *p value* |
|---|---|---|---|
| *NK cell($CD3^-CD16^+CD65^+$)* | moderate-F vs. | 195.33(100.22-290.44) | 0.7652 |
| | moderate-M | 189.56 (96.46 -282.65 ) | |
| | severe-F vs. | 247.50 (81.43 -413.57 ) | 0.7524 |
| | moderate-F | 195.33 (100.22 -290.45 ) | |
| *Th cell($CD3^+CD4^+$)* | moderate-F vs. | 626.44 (350.47 -902.42 ) | 0.0121* |
| | moderate-M | 396.56 (229.05 -564.06 ) | |
| | severe-F vs. | 407.40 (90.66 -724.14 ) | 0.202 |
| | moderate-F | 626.44 (350.47 -902.42 ) | |
| | severe-F vs. | 407.40 (90.66 -724.14 ) | 0.0571 |
| | severe-M | 140.33 (66.26 -214.40 ) | |
| | severe-M vs. | 140.33 (66.26 -214.40 ) | 0.0364* |
| | moderate-M | 396.56 (229.05 -564.06 ) | |
| | severe-F vs. | 407.40 (90.66 -724.14 ) | 0.202 |
| | moderate-F | 626.44 (350.47 -902.42 ) | |
| | moderate-F vs. | 626.44 (350.47 -902.42 ) | 0.0121* |
| | moderate-M | 396.56 (229.05 -564.06 ) | |
| *Ts cell($CD3^+CD8^+$)* | moderate-F vs. | 452.00 (292.81 -611.19 ) | 0.0533 |
| | moderate-M | 667.29 (436.73 -897.85 ) | |
| | severe-F vs. | 268.00 (119.01 -416.99 ) | 0.1007 |
| | moderate-F | 452.00 (292.81 -611.19 ) | |
| | severe-F vs. | 268.00 (119.01 -416.99 ) | 0.7429 |
| | severe-M | 231.33 (169.27 -293.40 ) | |
| | severe-M vs. | 231.33 (169.27 -293.40 ) | 0.0091** |
| | moderate-M | 667.29 (436.73 -897.85 ) | |
| *T cell（$CD3^+$）* | moderate-F vs. | 1110.67 (698.74 -1522.60 ) | 0.7065 |
| | moderate-M | 1030.10 (677.51 -1382.69 ) | |
| | severe-F vs. | 665.40 (230.14 -1100.66 ) | 0.1089 |
| | moderate-F | 1110.67 (698.74 -1522.60 ) | |

Cell counts of lymphocyte subset of patients with COVID-19, classified into 4 groups according to the disease severity, moderate (male=10,female=18) and severe (male=3,female=5), and patient sex, male (M) and female (F). Comparisons were done by the Mann-Whitney U test.

Table S5 The representative comparison of features of cell proportions of lymphocyte subset in COVID-19 patients classified by disease severity and sex followed by the mean, SD and p-value

| | | *Mean ±SD* | *p value* |
|---|---|---|---|
| NK cell($CD3^-CD16^+CD56^+$) | *moderate-F vs.* | *13.42 ((1.06-27.89)* | *0.3724* |
| | *moderate-M* | *13.23 (7.12 -19.34)* | |
| | *severe-F vs.* | *20.30 (5.46 -35.14)* | *0.6095* |
| | *moderate-F* | *13.42 ((1.06-27.89)* | |
| | *severe-F vs.* | *20.30 (5.46 -35.14)* | *0.6286* |
| | *severe-M* | *24.30 (20.00 -28.60)* | |
| Th cell($CD3^+CD4^+$) | *moderate-F vs.* | *41.50 (32.27 -50.73)* | *0.0056**** |
| | *moderate-M* | *26.43 (15.34 -37.51)* | |
| | *severe-F vs.* | *41.91 (31.20 -52.61)* | *0.7902* |
| | *moderate-F* | *41.50 (32.27 -50.73)* | |
| Ts cell($CD3^+CD8^+$) | *moderate-F vs.* | *30.62 (25.08 -36.15)* | *0.0314** |
| | *moderate-M* | *42.04 (28.80 -55.27)* | |
| | *severe-F vs.* | *26.22 (17.12 -35.32)* | *0.3021* |
| | *moderate-F* | *30.62 (25.08 -36.15)* | |
| Th cell($CD3^+CD4^+$)/Ts cell($CD3^+CD8^+$) | *moderate-F vs.* | *1.40 (0.96 -1.85)* | *0.0131** |
| | *moderate-M* | *0.77 (0.24 -1.29)* | |
| | *severe-F vs.* | *1.90 (1.29 -2.51)* | *0.1874* |
| | *moderate-F* | *1.40 (0.96 -1.85)* | |

Cell proportions of lymphocyte subset of patients with COVID-19, classified into 4 groups according to the disease severity, moderate (male=10,female=18) and severe (male=3,female=5), and patient sex, male (M) and female (F). Comparisons were done by the Mann-Whitney U test.
